# An Integrated Navigation Method Aided by Position Correction Model and Velocity Model for AUVs

**DOI:** 10.3390/s24165396

**Published:** 2024-08-21

**Authors:** Pengfei Lv, Junyi Lv, Zhichao Hong, Lixin Xu

**Affiliations:** 1 Ocean College, Jiangsu University of Science and Technology, Zhenjiang 212003, China; lvpengfei@just.edu.cn (P.L.); hongzhichao@just.edu.cn (Z.H.); 2 Shenzhen Institute for Advanced Study, University of Electronic Science and Technology of China, Shenzhen 518110, China; 202222280319@std.uestc.edu.cn; 3 Jiangsu Marine Technology Innovation Center, Nantong 226199, China

**Keywords:** autonomous underwater vehicle, underwater navigation, position correction model, velocity model, extended kalman filter

## Abstract

When autonomous underwater vehicles (AUVs) perform underwater tasks, the absence of GPS position assistance can lead to a decrease in the accuracy of traditional navigation systems, such as the extended Kalman filter (EKF), due to the accumulation of errors. To enhance the navigation accuracy of AUVs in the absence of position assistance, this paper proposes an innovative navigation method that integrates a position correction model and a velocity model. Specifically, a velocity model is developed using a dynamic model and the Optimal Pruning Extreme Learning Machine (OP-ELM) method. This velocity model is trained online to provide velocity outputs during the intervals when the Doppler Velocity Log (DVL) is not updating, ensuring more consistent and reliable velocity estimation. Additionally, a position correction model (PCM) is constructed, based on a hybrid gated recurrent neural network (HGRNN). This model is specifically designed to correct the AUV’s navigation position when GPS data are unavailable underwater. The HGRNN utilizes historical navigation data and patterns learned during training to predict and adjust the AUV’s estimated position, thereby reducing the drift caused by the lack of real-time position updates. Experimental results demonstrate that the proposed VM-PCM-EKF algorithm can significantly improve the positioning accuracy of the navigation system, with a maximum accuracy improvement of 87.2% compared to conventional EKF algorithms. This method not only improves the reliability and accuracy of AUV missions but also opens up new possibilities for more complex and extended underwater operations.

## 1. Introduction

Autonomous underwater vehicles [1] are important equipment for human development and the exploration of the ocean. They can accurately obtain underwater information such as seabed topography, marine meteorology, and hydrogeology. They can work underwater for a long time, which is safe, environmentally friendly, and efficient. A high-precision navigation system is essential [2] for the safe operation and recovery of AUVs, and has been one of the fundamental challenges in the development of autonomous underwater vehicles [3]. Sensor technology and navigation technology are the keys to determining the accuracy of the navigation system [4]. In recent years, deep neural networks [5], such as convolutional neural networks [6] and recurrent neural networks [7], have made great achievements in the fields of speech recognition, machine translation, target recognition, and so on. The problem of navigation based on machine learning [8] or deep learning [9] has also attracted the attention of scholars.

Many researchers have improved navigation accuracy [10] by enhancing the accuracy of sensor data. This accuracy enhancement is mainly carried out using the data from sensors such as the attitude and heading reference system (AHRS) [11], Doppler velocity log (DVL) [12], global positioning system (GPS) [13], and others.

Addressing the fact that the AHRS has a large measurement error of the pitch angle in rapidly changing environments such as near the water’s surface, Song [14] proposed a neural network-based approach for AUV navigation, which obtains a pitch angle with higher accuracy through the training and prediction of the neural network model; then, the navigation accuracy can be improved through dead reckoning. Karmozdi [15] constructed a three-degrees-of-freedom (3-DOF) AUV rotational motion dynamics model to assist the navigation system, introducing two Kalman filters to integrate the output of the auxiliary sensors and IMU to provide the best estimate. Addressing the fact that DVL data are prone to interference during AUV underwater navigation, resulting in no velocity data output for a short period, Cohen [16] proposed an end-to-end deep learning model, BeamsNet, using inertial data and previous measurements from DVL. This model is used to regress the current velocity of the AUV when no data are available from DVL. Cohen [17] proposed other data-driven methods, BeamsNetV1 and BeamsNetV2. BeamsNetV1 estimates DVL velocities based on a regression of beam measurements and inertial data at the current moment. BeamsNetV2 estimates DVL velocities based on a regression of DVL measurements at the current and past n moments. Lv [18] proposed an adaptive filtering method based on conventional navigation algorithms, DR, EKF, and iSAM, to solve the problem of GPS drift caused by the GPS antenna being easily covered when the AUV is sailing near the sea’s surface. This method can effectively improve the accuracy of the GPS surface by comparing the distance between the GPS position and the navigation position with the threshold, combined with the GPS failure time.

Improving the accuracy of sensor data can significantly enhance the navigation precision of AUVs [19]. However, the key to fundamentally improving navigation accuracy lies in the research and optimization of underwater navigation algorithms. These methods [20] are primarily categorized into three types: Kalman filter-based methods, particle filter-based methods, and graph optimization-based methods.

Kalman filter-based [21] navigation algorithms provide optimal estimates when the system adheres to the Gaussian distribution assumption and exhibits low nonlinearity. However, their performance may be limited when dealing with highly nonlinear systems. Particle filter [22] algorithms overcome the Gaussian distribution constraint of Kalman filters, making them suitable for systems with strong nonlinearity. They offer better adaptability and robustness, but their effectiveness in practical applications can be hindered by issues such as high computational cost, particle degeneration, and filter divergence. Graph optimization-based [23] algorithms build graph models using sensor data and solve nonlinear least squares problems to achieve global optimization. These algorithms are well-suited for large-scale environments in AUV navigation. However, during the linearization process of the nonlinear model, inevitable linearization errors can arise, impacting navigation accuracy. The navigation errors in these algorithms primarily stem from the lack of precise position information. Therefore, position aiding is crucial for enhancing the navigation accuracy of AUVs.

When autonomous underwater vehicles perform tasks underwater, they face significant challenges in maintaining accurate navigation due to the absence of GPS position assistance [24,25]. Unlike surface operations, where GPS can provide continuous position updates, underwater environments cause traditional navigation systems [26], such as the extended Kalman filter [27], to suffer from accumulated errors over time. This error accumulation leads to a gradual degradation in positioning accuracy, which can severely impact the effectiveness of AUV missions, especially those requiring precise maneuvering or long-duration deployments. To address these challenges and enhance the navigation accuracy of AUVs in environments where GPS data are unavailable, researchers carried out a series of studies [28].

Guo [29] proposed a nonlinear state reconstruction neural network that can reconstruct the state between two adjacent valid readings of an ultra-short baseline positioning system in real-time. This network estimates the position of an AUV based on an online process model. Mu [30] developed a navigation system model using a recurrent neural network (RNN) [31] framework to address the issue of inconsistent sensor data update frequencies. They employed both unidirectional and bidirectional Long Short-Term Memory (LSTM) networks to train GPS and other sensor data, resulting in end-to-end position data output. Lv [32] leveraged navigation data from periods with position aiding and constructed an error-corrected data set through graph optimization. They selected a hybrid gated recurrent neural network model, which aligns with the characteristics of time-series data, to develop an end-to-end position correction model, significantly improving navigation system accuracy. Although these methods effectively enhance the position accuracy of underwater robots using machine learning and deep learning techniques, challenges such as low output frequency and a narrow focus on specific issues remain.

This paper introduces an innovative navigation method that integrates a position correction model and a velocity model. The core idea is to mitigate the limitations of existing systems by incorporating additional predictive and corrective mechanisms into the navigation process. Since the update rate of DVL cannot meet the navigation requirements, we construct an online velocity model for velocity output when DVL is not updated. At the same time, since the AUV position error shows a divergent trend, that is, the accuracy of historical data is always higher than the current moment. Based on the timing characteristics of the navigation data, this paper selects the hybrid gated recurrent neural network (HGRNN) model to learn the historical navigation data containing GPS data, and constructs an offline position correction model for position assistance when there are no GPS data. The main contributions of this paper are summarized as follows:

1. Since DVL velocities are updated at a frequency of 1 Hz, and the update rate of the AUV navigation system is 10 Hz, this paper constructs a velocity model based on the constructed 3-degrees-of-freedom (3-DOF) dynamics model using the optimal pruning extreme machine learning method. When the DVL velocity is valid, its velocity is used for velocity model training and navigation computation; otherwise, the output of the velocity model is used to replace the DVL velocity for navigation calculations.
2. Since the AUV cannot obtain GPS longitude and latitude information for position assistance when navigating underwater, this paper constructs a position correction model based on a hybrid gated recurrent neural network to learn the correction of the navigation system when the AUV has position assistance. The EKF-PCM and iSAM-PCM models are constructed by GPS position correction of the EKF and iSAM methods, respectively. The output of the position correction model is used for auxiliary positioning during underwater navigation.
3. An extended Kalman filter, assisted by the velocity model and the position correction model, is constructed. The velocity model output is used for navigation during the DVL update interval. The GPS position is added to the observation vector for position assistance when there is an update to the GPS data. Otherwise, the displacement output by the position correction model is added to the observation vector for position assistance.

The structure of this paper is organized as follows: Section 2 provides a comprehensive description and analysis of the sensors involved in the navigation system, as well as the proposed algorithms. In Section 3, a series of experiments are designed based on the proposed algorithms, and the corresponding results are presented. Section 4 offers a detailed analysis of these experimental results. Finally, Section 5 provides an overall review of the paper, highlights the limitations of the algorithms, and discusses potential future research directions.

## 2. Materials and Methods

This section will mainly introduce the navigation system’s onboard sensors and their corresponding parameters, which are used in this paper. At the same time, the navigation system method used in this paper is introduced, from the general overview to the description of each key algorithm.

### 2.1. AUV Onboard Sensors of Navigation Systemlabel

The onboard navigation system sensors in this paper are the attitude and heading reference system (AHRS), Doppler velocity log (DVL), a global positioning system (GPS), and an intelligent pressure system (IPS). The specifications of the onboard sensors for the AUV navigation system are shown in Table 1.

- The AHRS, specifically the Ellipse-A AHRS, is produced by the French company SBG Systems, which provides the navigation system with heading, roll, pitch, three-axis acceleration, and three-axis angular velocity. The three-dimensional attitude angle is the key data that the navigation system can use to position the AUV.
- The DVL, specifically the NavQuest 600 micro DVL, comes from LinkQuest in the United States, which provides three-dimensional bottoming velocity and bottom height for navigation systems. The velocity data can be detected when the bottom height is 0.3–100 m. When the range is exceeded, the DVL sensor is unavailable.
- The GPS comes from the Swiss company U-Blox, whose specific model is the LEA-M8T GPS. When the field is sufficiently open and there are enough satellites, the GPS can provide latitude and longitude information without cumulative error. It needs to be converted to UTM coordinates to be used for AUVs. Due to the rapid attenuation of radio signals as they travel, the AUV cannot obtain position data from the GPS when it travels underwater, but the AUV can use GPS data to correct positioning errors when it sails on the surface.
- The specific model of the IPS is miniIPS, which comes from Valeport in the UK and provides the navigation system with the current depth of the AUV.

**Figure 1:**
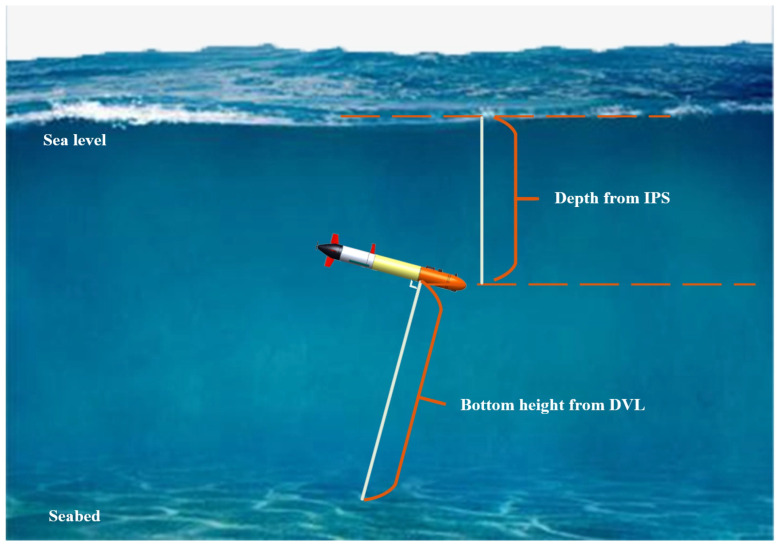
Detail of the depth and bottom height.

It should be noted here that, as shown in Figure 1, the depth from the IPS is the distance between the current position of the AUV and the sea level, and the bottom height from the DVL is the distance vertically downward to the seabed in the current attitude of the AUV.

### 2.2. Proposed Method for AUV Navigation

We propose an integrated navigation method based on the assistance of the position correction model and the velocity model. We will introduce the overall structure of the proposed navigation algorithm and then give the specific process and steps for the velocity model and the position correction model and its assistance to the EKF.

#### 2.2.1. Architecture of Proposed Method

**Figure 2:**
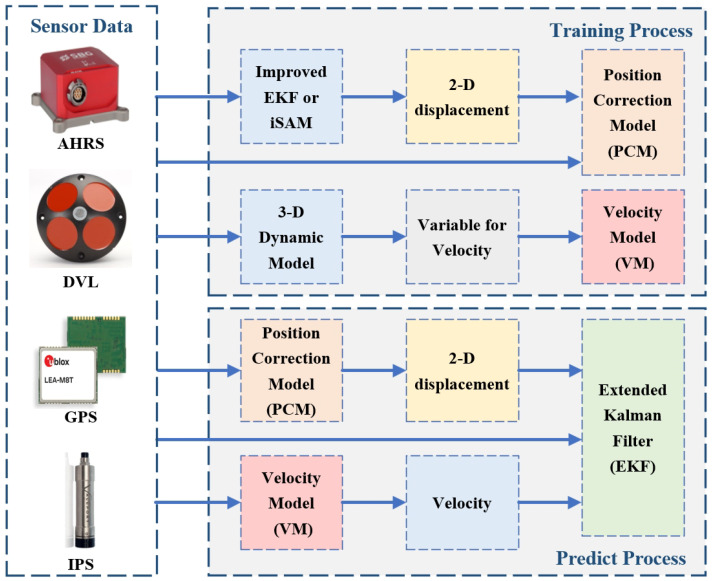
Architecture of the proposed underwater navigation framework for AUVs.

The architecture of the proposed underwater navigation framework for AUVs is illustrated in Figure 2. The data used are mainly from the AHRS, DVL, GPS, and IPS mentioned in Section 2.1. The proposed method is mainly divided into two processes: training and prediction.

The training process focuses on the construction of the position correction model and the velocity model. The velocity model is mainly used for the velocity output of the DVL velocity update interval. Since the velocity update frequency of the DVL is lower than the positioning update frequency of the navigation system, the AHRS based on the MEMS gyroscope has low accuracy and cannot provide velocity information, so it is proposed to build a velocity model to increase the frequency of the velocity data. The training process for the velocity model is as follows:

- The 3-D dynamics model is constructed based on the torpedo shape and the cross rudder structure of the AUV. The corresponding input variables are selected, and the output variables are provided by the updated DVL velocity.
- When there is an update of the DVL velocity and the bottoming depth is within the range, the values of the input variables confirmed by the dynamic model at the current moment and the values of the DVL bottoming velocity constitute a set of data, which is trained online by the OP-ELM.
- In order to ensure the training accuracy of the velocity model, multiple groups can be trained at the same time to form the required velocity model by weighted average.

Since the radio signal attenuates rapidly underwater, the GPS cannot obtain the position underwater, and low-cost AUVs are generally not equipped with sensors such as USBL. Therefore, it is proposed to construct a position correction model for position assistance during underwater navigation. The training process of the position correction model is as follows:

- Due to the update frequency of GPS being lower than that of the navigation system and the position drift occurring easily when the GPS antenna is covered, it is necessary to build a data set in advance.
- The input of the data set is provided by the sensors presented in Section 2.1. The data from the GPS position on the surface is combined with EKF or iSAM to perform a position solution. Then, we can obtain two-dimensional displacement data with uniform position correction.
- The sensor data and the processed two-dimensional displacement data are input into the HGRNN model for offline training to form a position correction model.

The prediction process is mainly based on the constructed velocity model and the position correction model to output the velocity and position, respectively. It is used to provide velocity during the DVL update intervals and displacement information when the AUV is navigating underwater without GPS data, and is input into the EKF for navigation. The prediction process of the proposed navigation method is as follows:

- Based on the update frequency of the navigation system, when the sensor data are updated, they are input into the EKF for the navigation calculation.
- When the DVL velocity is updated and the current bottom depth is within the range, the DVL velocity is used for navigation calculations. Otherwise, the output of the latest velocity model is used for the navigation calculation.
- When the GPS data are updated and the current depth of the AUV is within the threshold range, the GPS position is used for assisted navigation. When the AUV is sailing underwater, if the position correction model has velocity output, the assisted navigation is carried out based on the position of the PCM.

#### 2.2.2. Velocity Model

**Figure 3:**
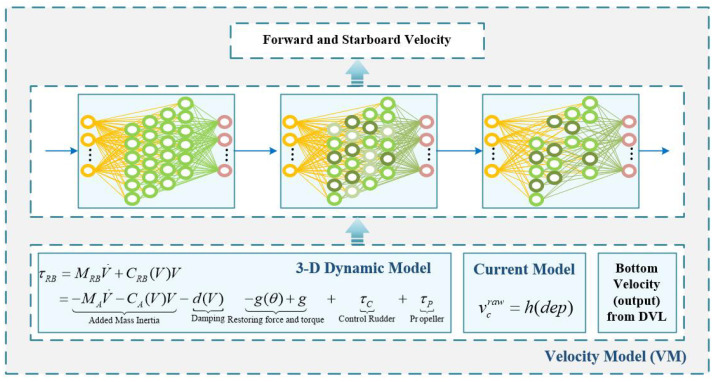
The flowchart of the velocity model.

In order to improve the update rate of the velocity to meet the requirements of the navigation system, a virtual velocity is constructed using the VM model for the output when the DVL is not updated. The flowchart of the velocity model is depicted in Figure 3.

Before constructing the velocity model, a 3-DOF dynamics model [18], shown in Equations (1) and (2), was constructed based on the shape and the crossover rudder structure of the AUV.

(1) $$\begin{array}{cc} X= & X_{\dot{u}}\dot{u}+\left(X_{u}+X_{\left|u\right|u}\left|u\right|\right)u-\left(Y_{\dot{v}}v+Y_{\dot{r}}r\right)r+X_{\delta \delta uu}\left(\delta r_{t}^{2}+\delta r_{b}^{2}+\delta e_{p}^{2}+\delta e_{s}^{2}\right)u^{2}+X_{b}\\ \; & +X_{vr}vr+X_{vv}v^{2}+X_{rr}r^{2}+\left(B-W\right)sin\left(\xi_{y}\right)+X_{\left|n\right|u}\left|n\right|\left(1-w\right)u+X_{\left|n\right|n}\left|n\right|n\\ Y= & Y_{\dot{v}}\dot{v}+Y_{\dot{r}}\dot{r}+\left(Y_{v}+Y_{\left|v\right|v}\left|v\right|\right)v+\left(Y_{r}+Y_{\left|r\right|r}\left|r\right|+X_{\dot{u}}u\right)r+Y_{\delta uu}\left(\delta r_{t}+\delta r_{b}\right)u^{2}+Y_{b}\\ \; & +Y_{\delta uv}uv+Y_{uv}uv+Y_{\delta ur}ur+Y_{ur}ur-\left(B-W\right)cos\left(\xi_{y}\right)sin\left(\xi_{x}\right)\\ N= & N_{\dot{v}}\dot{v}+N_{\dot{r}}\dot{r}+\left(Y_{\dot{v}}v+Y_{\dot{r}}r\right)u+\left(-X_{\dot{u}}u+N_{v}+N_{\left|v\right|v}\left|v\right|\right)v+\left(N_{r}+N_{\left|r\right|r}\left|r\right|\right)r+N_{b}\\ \; & +N_{\delta uu}\left(\delta r_{t}+\delta r_{b}\right)u^{2}+N_{\delta uv}uv+N_{uv}uv+N_{\delta ur}ur+N_{ur}ur \end{array}$$

(2) $$\begin{array}{cc} X= & m\dot{u}-mvr\\ Y= & m\dot{v}+mur\\ \; & N=I_{z}\dot{r} \end{array}$$

where

*m*: the mass of the AUV in liquid.
$I_{z}$: the rigid body moment of inertia generated around the *z*-axis.
*n*, *w*: the propeller rotation rate and the wake fraction number.
*W*, *B*: the weight and the buoyancy of the AUV in the same liquid.
$\xi_{x}$, $\xi_{y}$: the attitude of roll and pitch.
$\delta r_{t}$, $\delta r_{b}$: the deflection angles of the top and bottom rudders.
$\delta e_{p}$, $\delta e_{s}$: the deflection angles of port and starboard elevators.
*u*, *v*, *r*: the *x*-forward velocity, *y*-starboard velocity, and *z*-downward angular velocity.
$\dot{u}$, $\dot{v}$, $\dot{r}$: the *x*-forward acceleration, *y*-starboard acceleration, and *z*-downward angular acceleration.
*X*, *Y*, *N*: the axial force, lateral force, and yawing external moment on the AUV.
$X_{\left(\cdot \right)}$, $Y_{\left(\cdot \right)}$, $N_{\left(\cdot \right)}$: hydrodynamic coefficients.

After solving the values of $X_{\left(\cdot \right)}$, $Y_{\left(\cdot \right)}$, $N_{\left(\cdot \right)}$, input the corresponding sensor values into Equations (1) and (2) to obtain the water-relative velocities *u* and *v*. Assuming that the magnitude and direction of the current velocity remain unchanged in a short period of time, the current velocity $V_{c}^{n}=\left[u_{c}^{n},v_{c}^{n}\right]$ depends only on the depth. In order to add the current velocity to the water-relative velocity, it needs to be rotated to the AUV coordinate system, which is shown in Equation (3).

(3) $$\begin{array}{cc} u_{c}^{b}= & u_{c}^{n}cos\xi_{z}+v_{c}^{n}sin\xi_{z}\\ v_{c}^{b}= & -u_{c}^{n}sin\xi_{z}+v_{c}^{n}cos\xi_{z} \end{array}$$

where $u_{c}^{n}$ and $v_{c}^{n}$ represent the current velocity northward and eastward, respectively, $u_{c}^{b}$ and $v_{c}^{b}$ represent the current velocity forward and starboard in the AUV coordinate system, and $\xi_{z}$ is the heading angle.

Through the above formula, the variable related to the base velocity is obtained, which is taken as the input variable $x_{i}=\left[u,v,\dot{r},\xi_{x},\xi_{y},\xi_{z},\delta r_{t},\delta r_{b},\delta e_{p},\delta e_{s},n,dep\right]$, and the forward and starboard velocity of DVL is taken as the output variable $y_{i}$ to construct the data set. ($x_{i}$, $y_{i}$) is the ith data.

When the DVL velocity is valid, the data set is continuously expanded according to the above format, and then the corresponding data are constructed by an extreme learning machine (ELM) such as Equation (4).

(4) $$\left[\begin{array}{ccc} g(w_{1}\cdot x_{1}+b_{1}) & \cdots & g(w_{N}\cdot x_{1}+b_{N})\\ \vdots & \cdots & \vdots \\ g(w_{1}\cdot x_{M}+b_{1}) & \cdots & g(w_{N}\cdot x_{M}+b_{N}) \end{array}\right]\left[\begin{array}{c} \beta_{1}^{T}\\ \vdots \\ \beta_{N}^{T} \end{array}\right]=\left[\begin{array}{c} y_{1}^{T}\\ \vdots \\ y_{M}^{T} \end{array}\right]$$

where $w_{i}$ and $b_{i}$ represent the input weights and bias connecting the *i*th hidden neurons and input neurons, respectively, and $\beta_{i}$ is the output weights connecting the *i*th hidden neurons and output neurons.

In order to improve the fitting effect, multiresponse sparse regression (MRSR) is used to rank the importance of the hidden layer neurons, and then the let-one-out (LOO) criterion is used to remove less important ones to form an optimal pruning extreme learning machine [33]. After online training, the corresponding parameters can be obtained to form the velocity model.

#### 2.2.3. Position Correction Model

Since the error of the navigation system increases over time, a position correction model is constructed to learn historical time series data, which mainly provides position information for AUV underwater navigation without GPS. The premise for using this model is that the data come from the same AUV and the sensor mounting position inside the AUV has not moved. Otherwise, the data set needs to be reconstructed and the new model trained. Since the update rate of the GPS data is lower than that of the navigation system, and the GPS has occasional drifts, the pre-processing of GPS position correction based on EKF or iSAM before construction can obtain two-dimensional displacement data with the same update rate as the navigation system. The algorithm steps are outlined in [32].

Based on the above data, the data set of the position correction model is constructed. The input vector is represented as $I^{PCM}=\left[\xi_{x},\xi_{y},\xi_{z},v_{x},v_{y},v_{z},a_{x},a_{y},a_{z},w_{x},w_{y},w_{z}\right]$, and the output vector is represented as $O^{PCM}=\left[\delta_{x}^{VM},\delta_{x}^{VM}\right]$, where h,p,r are the roll, pitch, and yaw angle, respectively; $v_{x},v_{y},v_{z}$ are the forward, starboard, and downward velocities in the AUV coordinate system, respectively; $a_{x},a_{y},a_{z}$ are the forward, starboard and downward accelerations respectively; and $w_{x},w_{y},w_{z}$ are the three-axis angular velocities, respectively. $\delta_{x},\delta_{y}$ denote the calculated north and east displacements, respectively.

**Figure 4:**
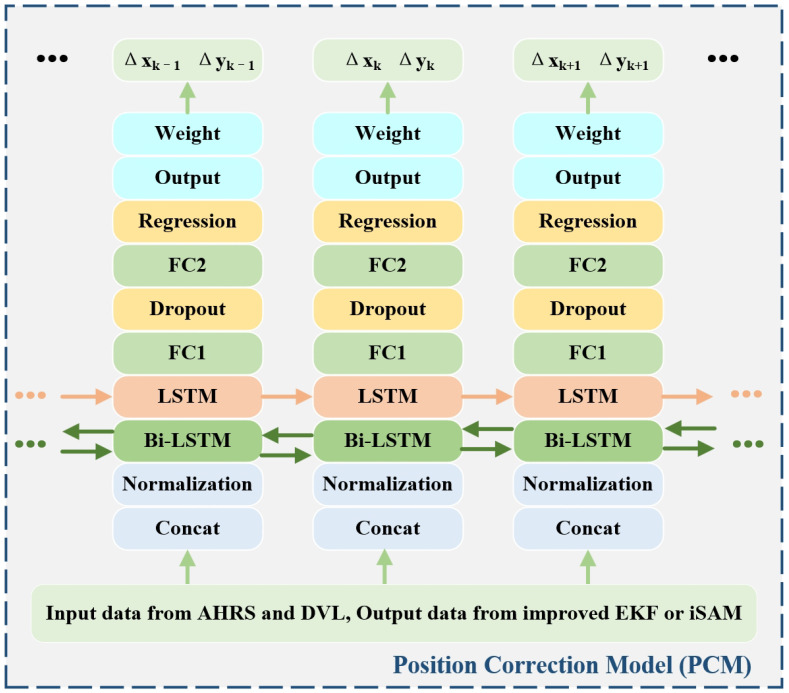
The flowchart of the position correction model.

After constructing the data set, use the position correction model shown in Figure 4 for training. The corresponding data are connected and normalized to obtain the input sequence. Then input it into the hybrid recurrent neural network (HGRNN) composed of bi-LSTM and LSTM, where the output mode of Bi-LSTM is “sequence” and the output mode of LSTM is “last”. After that, two fully connected layers, FC1 and FC2, are used. To prevent data overfitting, FC1 is followed by a random inactivation layer, while FC2 is followed by a regression layer to obtain the normalized output. After assigning the initial weight, the displacement data can be obtained as output. Where the PCM uses the Adam algorithm for model optimization, the initial learning rate of the model is 0.001, and the loss function is provided by the root-mean-square error (RMSE). The position correction model can be obtained by training based on the above parameters.

#### 2.2.4. Proposed Algorithm VM-PCM-EKF

In this paper, we propose an underwater navigation method based on the assistance of the velocity model and position correction model, which improves the update rate of the velocity data through the velocity model, and provides position data for AUV underwater navigation through the position correction model to improve the accuracy of the navigation system. The steps of the algorithm are shown in Algorithm 1.

After the navigation system is turned on, the state vector $X_{0}$ and the covariance matrix $P_{0}$ are initialized. At the same time, the position of the initial point $UTM_{0}^{GPS}$ is obtained by converting the current GPS longitude and latitude coordinates into Universal Transverse Mercator Grid System (UTM) coordinates.

If there is a data update, start predicting the state vector $X_{k|k-1}$ at time *k* and the covariance matrix $P_{k|k-1}$ as shown in Equation (5).

(5) $$\begin{array}{c} X_{k|k-1}=f\left(X_{k-1},O_{k-1}\right)\\ P_{k|k-1}=F_{k-1}P_{k-1}F_{k-1}^{T}+Q_{k-1} \end{array}$$

where $X_{k-1}$ is the state vector at time k−1, $O_{k-1}$ is Gaussian noise with mean zero, and covariance matrix $Q_{k}$, $F_{k-1}$ is the Jacobi matrix of the motion function f(·) on the state vector $X_{k-1}$.

(6) $$\begin{array}{c} X_{k-1}={\left[x_{k-1},y_{k-1},v_{x,k-1},v_{y,k-1},a_{x,k-1},a_{y,k-1},\xi_{z,k-1},w_{z,k-1}\right]}^{T}\\ O_{k-1}={\left[O_{x,k-1},O_{y,k-1},O_{vx,k-1},O_{vy,k-1},O_{ax,k-1},O_{ay,k-1},O_{\xi z,k-1},O_{wz,k-1}\right]}^{T} \end{array}$$

Then, the measurement update step is performed according to the data update of the sensor. We select different observation vectors according to the updates of DVL and GPS while the AUV is sailing on the surface. Depending on the availability of location data, the observation vectors are divided into $Z_{1k}$ and $Z_{2k}$ as shown in Equation (7).

(7) $$\begin{array}{c} Z_{1k}={\left[x_{k},y_{k},v_{x,k},v_{y,k},a_{x,k}^{AHRS},a_{y,k}^{AHRS},\xi_{z,k}^{AHRS},w_{z,k}^{AHRS}\right]}^{T}\\ Z_{2k}={\left[v_{x,k},v_{y,k},a_{x,k}^{AHRS},a_{y,k}^{AHRS},\xi_{z,k}^{AHRS},w_{z,k}^{AHRS}\right]}^{T} \end{array}$$

where $x_{k}$ and $y_{k}$ denote the northward and eastward displacements, $v_{x,k}$ and $v_{y,k}$ denote the forward and starboard velocities, $a_{x,k}^{AHRS}$ and $a_{y,k}^{AHRS}$ denote the forward and starboard accelerations measured by AHRS, $\xi_{z,k}^{AHRS}$ denotes the bow angle measured by AHRS, and $w_{z,k}^{AHRS}$ denotes the angular velocity around the *z*-axis measured by AHRS.

Determine whether the AUV is navigating on the surface or underwater from its current depth. When the DVL data are updated, the velocities $v_{x,k}$ and $v_{y}$ of the observation vector are provided by DVL, shown in Equation (8); otherwise, $v_{x,k}$ and $v_{y}$ are provided by the output of the velocity model shown in Equation (9).

(8) $$\begin{array}{c} v_{x,k}=v_{x,k}^{DVL}\\ v_{y,k}=v_{y,k}^{DVL} \end{array}$$

(9) $$\begin{array}{c} v_{x,k}=v_{x,k}^{VM}\\ v_{y,k}=v_{y,k}^{VM} \end{array}$$

Meanwhile, the displacements $x_{k}$ and $y_{k}$ of the observation vectors as shown in Equation (10) are obtained from the conversion of GPS position, when there is an update of the GPS. Otherwise, the displacements of the observation vectors are null when the AUV is sailing on the surface.

(10) $$\begin{array}{c} x_{k}=UTM_{k}^{GPS}\left(x\right)-UTM_{0}^{GPS}\left(x\right)\\ y_{k}=UTM_{k}^{GPS}\left(y\right)-UTM_{0}^{GPS}\left(y\right) \end{array}$$

When the AUV navigates underwater, the velocity of the observation vectors is consistent with that when it is on the surface. Moreover, the displacement of the observation vectors as shown in Equation (11) are jointly determined by the outputs of the position correction model and the position at the previous moment.

(11) $$\begin{array}{c} x_{k}=X_{k-1}\left(1\right)+\delta_{x,k}^{VM}\\ y_{k}=X_{k-1}\left(2\right)+\delta_{y,k}^{VM} \end{array}$$

where $X_{k-1}\left(1\right)$ and $X_{k-1}\left(2\right)$ are the displacements northward and eastward for the AUV at time k−1, $\delta_{x,k}^{VM}$ and $\delta_{y,k}^{VM}$ are the single step displacements for the AUV at time *k*.

When the displacement data in the observation vector exist, $Z_{1k}$ is used as the observation vector, in which case the Jacobi matrix of the observation function is denoted by $H_{1k}$, which is an 8-dimensional unit matrix, as shown in Equation (12).

(12) $$\begin{array}{c} H_{1k}=I_{8\times 8} \end{array}$$

**Algorithm 1:** The proposed algorithm VM-PCM-EKF.

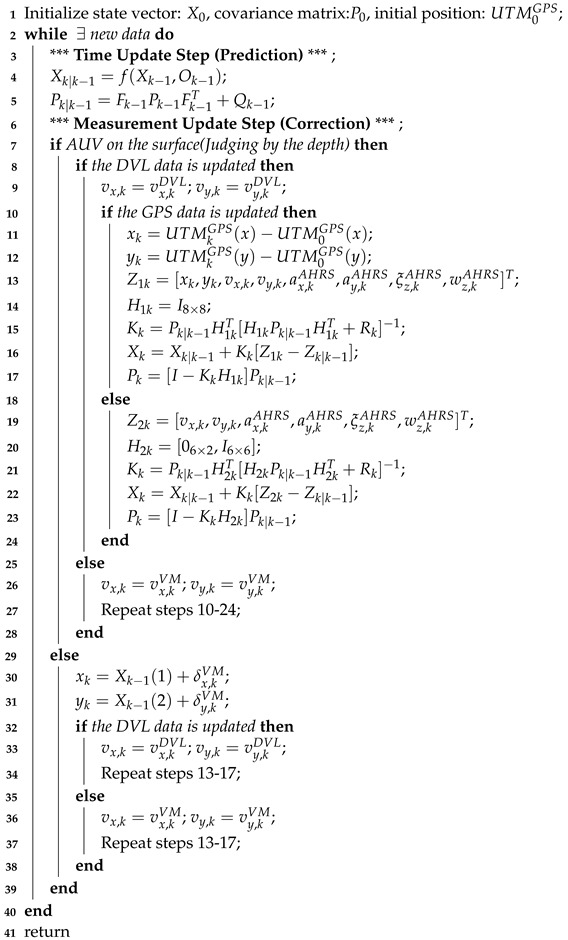



The corresponding Kalman gain $K_{k}$ is shown in Equation (13), where $R_{k}$ is the covariance matrix of the observation noise.

(13) $$\begin{array}{c} K_{k}=P_{k|k-1}H_{1k}^{T}{\left[H_{1k}P_{k|k-1}H_{1k}^{T}+R_{k}\right]}^{-1} \end{array}$$

At this point, the state vectors $X_{K}$ and covariance matrix $P_{K}$ at time *k* can be calculated as shown in Equation (14).

(14) $$\begin{array}{c} X_{k}=X_{k|k-1}+K_{k}\left[Z_{1k}-Z_{k|k-1}\right]\\ P_{k}=\left[I-K_{k}H_{1k}\right]P_{k|k-1} \end{array}$$

When displacement data are absent in the observation vector, $Z_{2k}$ is utilized as the observation vector. In this scenario, the Jacobian matrix of the observation function is represented by $H_{2k}$, as detailed in Equation (15).

(15) $$\begin{array}{c} H_{2k}=\left[0_{6\times 2},I_{6\times 6}\right] \end{array}$$

The corresponding Kalman gain $K_{k}$ is shown in Equation (16).

(16) $$\begin{array}{c} K_{k}=P_{k|k-1}H_{2k}^{T}{\left[H_{2k}P_{k|k-1}H_{2k}^{T}+R_{k}\right]}^{-1} \end{array}$$

At this point, the state vectors $X_{k}$ and covariance matrix $P_{k}$ at time *k* can be calculated as shown in Equation (17).

(17) $$\begin{array}{c} X_{k}=X_{k|k-1}+K_{k}\left[Z_{2k}-Z_{k|k-1}\right]\\ P_{k}=\left[I-K_{k}H_{2k}\right]P_{k|k-1} \end{array}$$

## 3. Experimental Results

To verify the effectiveness of the proposed algorithm, this section shows the experimental results mainly from three parts: the velocity model, the position correction model, and the proposed VM-PCM-EKF.

### 3.1. The Results of Velocity Model

The objective of this experiment is to evaluate the effectiveness of the velocity model in the AUV navigation system, specifically its performance in estimating forward velocity and starboard velocity. By comparing with the true velocity data provided by DVL, the experiment aims to verify the accuracy and reliability of the velocity model in filling the gaps during DVL update intervals. Specifically, the experiment will analyze the results from three aspects—velocity estimation, the mean (Error-Mea) and standard deviation (Error-Std) of step-by-step error, and root mean square error (RMSE)—to comprehensively assess the model’s performance in practical applications.

**Figure 5:**
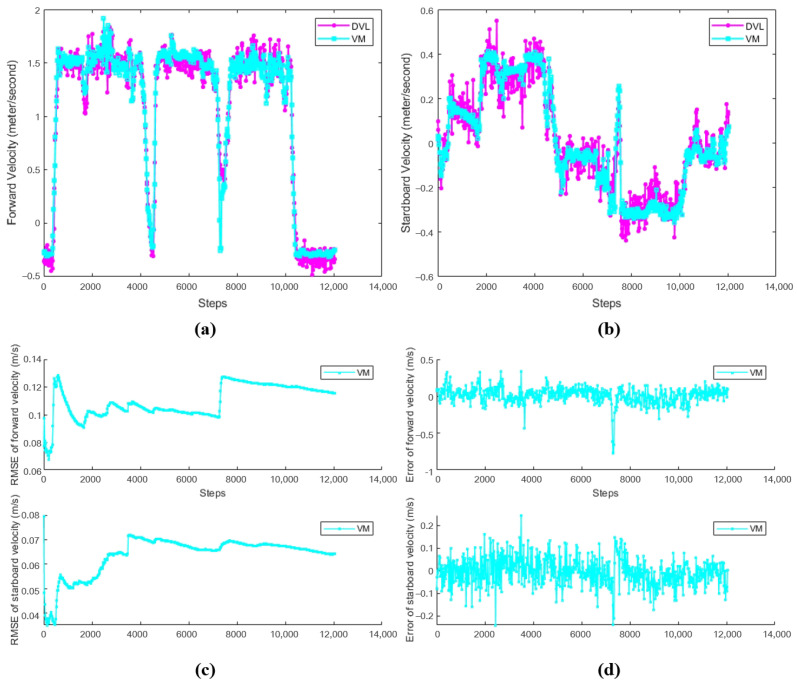
Comparison between DVL and velocity model. (**a**) Forward velocity. (**b**) Starboard velocity. (**c**) RMSE between VM and ground truth. (**d**) Error between VM and ground truth.

Figure 5 shows the comparison of DVL and the velocity model, where (a) and (b) represent the velocity comparison in the forward and starboard directions, respectively, and (c) and (d) represent the root mean square error and single step error between DVL and VM, respectively. The magenta line represents the velocity of the DVL as the ground truth, and the cyan line represents the output of the velocity model.

The detailed values of the velocity model and the DVL velocity in the the mean (Error-Mea) and standard deviation (Error-Std) of the single-step error and the overall root-mean-square error are shown in Table 2.

### 3.2. The Results of Position Correction Model

To validate the effectiveness of the position correction model in AUV navigation, this experiment evaluates the performance of PCM in estimating northward and eastward displacements. The PCM is assessed using two data sets constructed from different algorithms: the improved incremental smoothing and mapping (IiSAM) and the improved extended Kalman filter (IEKF). Specifically, PCM1 refers to the position correction model trained on the data set constructed with the IEKF, while PCM2 refers to the model trained on the data set built using the IiSAM algorithm. The experiment involves comparing the northward and eastward displacements estimated by the PCM with the true displacements. Additionally, the performance of both position correction models will be analyzed in terms of the mean and standard deviation of step-by-step errors, as well as the root mean square error. This analysis will provide a comprehensive assessment of the PCM’s effectiveness in improving navigation accuracy under varying conditions and help determine the relative performance of the two data sets used for model training.

**Figure 6:**
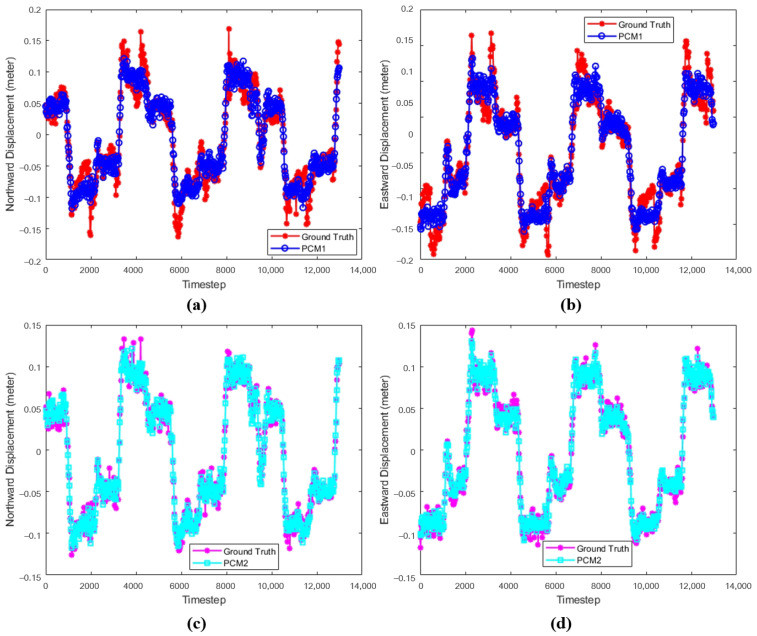
Comparison between position correction models and ground truth. (**a**,**b**) Northward and eastward displacements of PCM1. (**c**,**d**) Northward and eastward displacements of PCM2.

Figure 6 shows the comparison between PCM and ground truth, where (a) and (b) represent the comparison between PCM1 and the ground truth in the northward and eastward displacements, respectively, and (c) and (d) denote the comparison between PCM2 and the ground truth in the northward and eastward displacements, respectively. The red and magenta lines are the ground truth, and the green and blue lines indicate PCM1 and PCM2, respectively.

**Figure 7:**
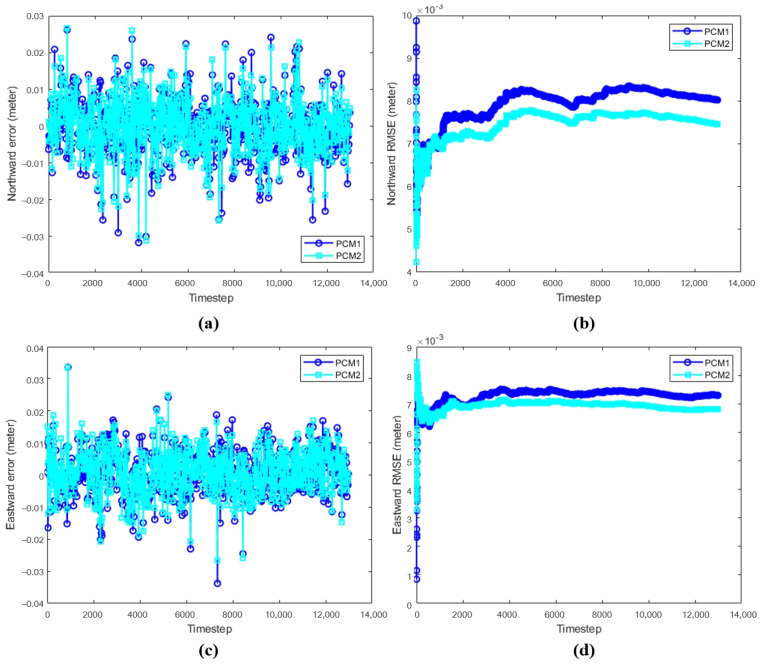
Comparison of error and RMSE between position correction models and ground truth. (**a**) Northward error. (**b**) Northward RMSE. (**c**) Eastward error. (**d**) Eastward RMSE.

Figure 7 shows the error and RMSE between the position correction models and the ground truth, where (a) and (c) represent the northward and eastward errors between the two position correction models and the true value, respectively, and (b) and (d) represent the northward and eastward root mean square errors, respectively.

The mean error (Error-Mea) and standard deviation (Error-Std) for northward and eastward displacements, as well as the root mean square error (RMSE) for the entire process, are detailed in Table 3. These data complement the results presented in Figure 7, providing a clearer presentation of the position correction model’s performance. By listing key data in the table, a more accurate comparison and interpretation of PCM’s effectiveness in practical applications can be achieved.

### 3.3. The Results of Proposed Method VM-PCM-EKF

This section aims to evaluate the enhancement effects of the velocity model and position correction model on the extended Kalman filter. The experiment uses GPS-corrected navigation data from the data set as the ground truth to analyze the performance improvements. Results are assessed in terms of trajectory accuracy, root mean square error, and single-step positional error at the endpoint to provide a comprehensive understanding of how VM and PCM contribute to the overall accuracy and reliability of the EKF in underwater navigation systems. “Distance” refers to the actual distance traveled by the AUV as shown in the results, while “Accuracy” denotes the ratio of the endpoint position’s single-step error to the actual traveled distance.

**Figure 8:**
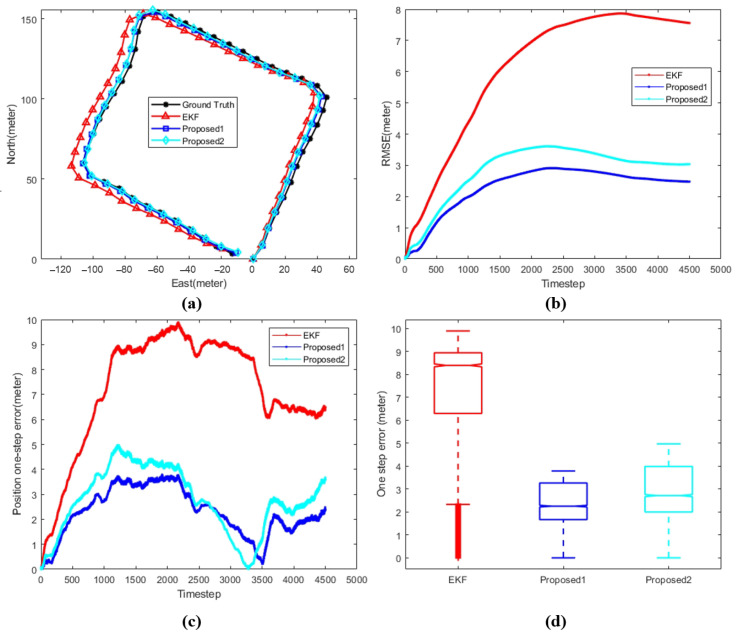
Comparison between different algorithms with respect to data 1. (**a**) AUV trajectory. (**b**) Position RMSE. (**c**) Position one-step error. (**d**) Position error statistics.

**Figure 9:**
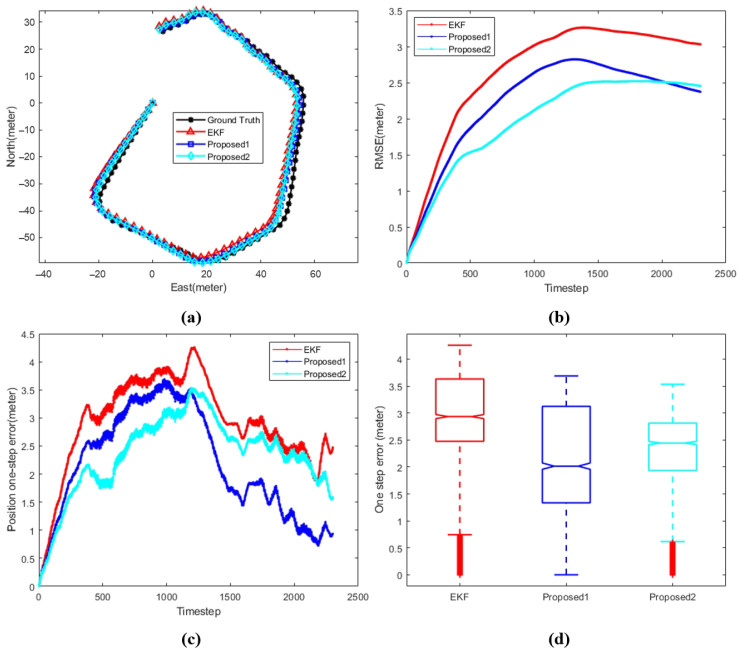
Comparison between different algorithms with respect to data 2. (**a**) AUV trajectory. (**b**) Position RMSE. (**c**) Position one-step error. (**d**) Position error statistics.

**Figure 10:**
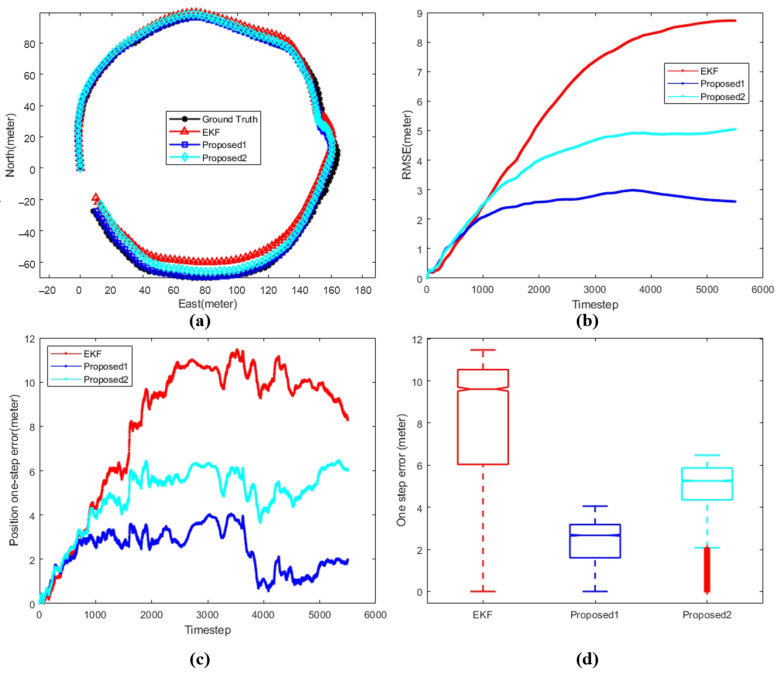
Comparison between different algorithms with respect to data 3. (**a**) AUV trajectory. (**b**) Position RMSE. (**c**) Position one-step error. (**d**) Position error statistics.

Figure 8, Figure 9 and Figure 10 shows the comparison between different algorithms, where (a) shows the trajectory comparison between various algorithms and ground truth, (b) shows the root mean square error between different algorithms and ground truth, (c) and (d) shows different representations of the single-step position error between various algorithms and ground truth. The black line represents the ground truth, the red line represents the extended Kalman filter (EKF), the blue line represents proposed1, where the data set of the position correction model is generated by the improved EKF, and the cyan line represents proposed2, where the data set of the position correction model is generated by the improved iSAM.

The navigation distance, RMSE, single-step error at the endpoint, median of single-step error (Error-Med), and positioning accuracy of different algorithms in the three sets of data are shown in Table 4.

## 4. Experimental Analysis

To comprehensively validate the effectiveness of the proposed algorithms, experiments were designed at three levels: velocity model, position correction model, and the combined VM-PCM-EKF navigation algorithm. The corresponding results are presented in Figure 5, Figure 6, Figure 7, Figure 8, Figure 9 and Figure 10 and Table 2, Table 3 and Table 4.

Figure 5 and Table 2 illustrate the comparison between the output of the velocity model and the DVL velocity. The experimental results demonstrate that the velocity model, based on dynamic modeling and the optimally pruned extreme learning machine, can effectively predict dynamic changes in velocity. Specifically, the mean error is 0.011 m/s for the forward velocity and 0.0035 m/s for the starboard velocity. This indicates that the VM output can be reliably used for navigation during the DVL update intervals, ensuring the continuity and reliability of the navigation system. Moreover, in scenarios where the DVL is out of range and unable to provide velocity output, the VM model can continue to provide reliable velocity estimates, preventing system failure and supplying the control system with high-frequency updates, which is conducive to precise autonomous control.

Figure 6 and Figure 7, along with Table 3, show the comparison between position correction models based on two different data sets. The experiments indicate that both PCM1 and PCM2 can accurately predict northward and eastward displacement. Specifically, the mean error of PCM1 in northward displacement is 0.006 m, while that of PCM2 is 0.0058 m, and the mean errors in eastward displacement are 0.0055 m and 0.0053 m, respectively. Overall, PCM2 slightly outperforms PCM1, further validating the model’s effectiveness in position correction under conditions lacking GPS signals. This contributes significantly to enhancing the precision of AUV navigation.

Figure 8, Figure 9 and Figure 10 and Table 4 display the performance of the proposed VM-PCM-EKF integrated navigation algorithm across three different data sets. Figure 8 shows the performance of the proposed algorithm in data 1. By analyzing the specific values in Table 4, it is evident that the two proposed algorithms significantly outperform the traditional EKF in terms of root mean square error (RMSE), endpoint position error, and median error. Notably, the PCM1-assisted EKF, which is trained using the IEKF, demonstrates superior performance compared to the IiSAM-based approach. The navigation accuracy improves from 0.014327 for the traditional EKF to 0.005254 with Proposed1, resulting in an accuracy enhancement of 87.2%.

Figure 9 shows the performance of the proposed algorithms on the second data set. By analyzing the specific values in Table 4, it is clear that both proposed algorithms significantly outperform the traditional EKF in terms of RMSE, end-point position error, and median error throughout the entire process. Although the PCM1-assisted EKF, which is trained using the improved EKF, exhibits a larger error during the initial stages of the trajectory compared to Proposed2, it ultimately achieves a better performance by the end of the trajectory. The navigation accuracy improves from 0.010466 with the traditional EKF to 0.003941 with Proposed1, reflecting an accuracy improvement of 62.34%.

Figure 10 illustrates the performance of the proposed algorithms on the third data set. By examining the values in Table 4, it is evident that both proposed algorithms outperform the traditional EKF significantly in terms of RMSE, endpoint position error, and median error. The overall effectiveness of Proposed1 is markedly superior to Proposed2, with navigation accuracy improving from 0.016747 with the traditional EKF to 0.003939 with Proposed1, representing a 76.5% increase in accuracy.

The experimental results comprehensively validate the significant impact of the proposed VM-PCM-EKF integrated navigation algorithm on improving the accuracy and reliability of AUV navigation. The VM model effectively fills the gap in velocity estimation during DVL update intervals, the PCM model provides accurate position correction, and the VM-PCM-EKF algorithm achieves a substantial enhancement in navigation accuracy over the traditional EKF. These methods show great potential in practical underwater navigation applications, offering more reliable technical support for executing complex underwater missions.

## 5. Conclusions

This paper addresses two critical issues in AUV navigation: the lower update rate of DVL compared to the navigation system’s update frequency, and the accumulation of navigation errors underwater due to the lack of GPS signal, which cannot be effectively corrected. To solve these problems, we propose an online velocity model that provides an accurate velocity during DVL data update gaps. Additionally, we developed an offline position correction model based on GPS data, which is used for position correction when the AUV is navigating underwater without GPS.

Experimental results demonstrate that the proposed integrated navigation algorithm VM-PCM-EKF significantly enhances the accuracy of the navigation system. While the performance of PCM1 is slightly inferior to PCM2 when analyzed individually, the PCM1 model, trained using data sets from the improved extended Kalman filter, shows a better performance when assisting EKF in positioning. Compared to conventional EKF algorithms, accuracy is improved by up to 87.2%.

However, the application of this method comes with certain limitations. First, it requires the AUV to have sufficient surface time with GPS to collect a large amount of high-quality data for model training. Furthermore, the effectiveness of this method on other underwater exploration devices remains to be validated. Future research will continue to explore the role of different deep learning models in navigation system correction to further improve accuracy and expand the applicability of this method.

## Figures and Tables

**Table 1:** Specifications of onboard sensors for AUV navigation system.

Sensors	Type	Parameter	Value
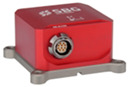		Heading	1° RMS
	Ellipse-A AHRS	Pitch	0.2° RMS
	SBG Systems	Roll	0.2° RMS
		Update Rate	10 Hz (up to 200 Hz)
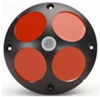		Velocity Accuracy 1 m/s	
	NavQuest 600 micro DVL	Altitude Range	0.3–110 m
	LinkQuest	Maximum Velocity	20 knot
		Update Rate	1 Hz
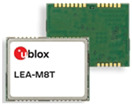		Position Accuracy	2.5 m CEP
	LEA-M8T GPS	Acquisition	GPS & BeiDou
	U-blox	Update Rate	1 Hz
		Cold Starts	25 s
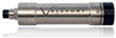	miniIPS	Accuracy	0.01% Range
	Valeport	Update Rate	1 Hz

**Table 2:** The numerical comparison of the velocity model.

Type	Error-Mea (m/s)	Error-Std (m/s)	RMSE (m/s)
Forward Velocity	0.011	0.0132	0.2699
Stardboard Velocity	0.0035	0.0041	0.0643

**Table 3:** The numerical comparison of two position correction models.

Algorithm	Northward Displacement (m)			Eastward Displacement (m)		
	Error-Mea	Error-Std	RMSE	Error-Mea	Error-Std	RMSE
PCM1	0.006	0.0054	0.008	0.0055	0.0048	0.0073
PCM2	0.0058	0.0047	0.0075	0.0053	0.0042	0.0068

**Table 4:** Numerical performance of different algorithms in three sets of data.

Data	Distance (m)	Algorithm	RMSE (m)	Error (m)	Error-Med (m)	Accuracy
Data 1	453	EKF	7.56	6.49	8.39	0.014327
		Proposed 1	2.48	2.38	2.26	0.005254
		Proposed 2	3.04	3.59	2.72	0.007925
Data 2	236	EKF	3.03	2.47	2.93	0.010466
		Proposed 1	2.37	0.93	2.01	0.003941
		Proposed 2	2.45	1.59	2.44	0.006737
Data 3	498	EKF	8.72	8.34	9.61	0.016747
		Proposed 1	2.6	1.96	2.67	0.003936
		Proposed 2	5.04	6.03	5.25	0.012108

## Data Availability

Data set available on request from the authors. The raw data supporting the conclusions of this article will be made available by the authors on request.
